# Intermediary inflammatory reaction after micropulse cyclophotocoagulation diode therapy: a case report

**DOI:** 10.1186/s13256-022-03307-9

**Published:** 2022-03-02

**Authors:** Gaëtane Ghion, Alexandra Singh, Sayeh Pourjavan

**Affiliations:** 1 Chirec Hospital Group Delta Hospital, Brussels, Belgium; 2 Cliniques Universitaires Saint-Luc, Brussels, Belgium

**Keywords:** Case report, Ophthalmology, Glaucoma, Micropulse, Vitritis

## Abstract

**Introduction:**

Of the many types of laser cyclophotocoagulation procedures, micropulse cyclophotocoagulation diode is praised as a noninvasive, safe, and effective procedure with few complications. In this case report, we describe a rare complication that, to the best of our knowledge, has not been previously reported.

**Case report:**

We report on the case of a 66-year-old African man with a history of end-stage primary open-angle glaucoma. One week after undergoing micropulse cyclophotocoagulation diode therapy in both eyes, he developed severe intermediary inflammation in one eye, associated with decreased visual acuity. The intraocular pressure had significantly decreased after the procedure and was well controlled with intraocular-pressure-lowering medications. Slit lamp examination revealed a moderate anterior chamber inflammation, anterior vitritis, and a large inflammatory membrane attached to the posterior surface of the intraocular implant. A vitrectomy was finally performed in the left eye because of the persistent intermediary inflammation despite the use of high doses of topical and subconjunctival corticosteroids.

**Conclusion:**

Intermediary uveitis is a rare complication after micropulse cyclophotocoagulation diode therapy. To the best of our knowledge, there have been no reports of vitritis after a noncomplicated micropulse cyclophotocoagulation diode in primary open-angle glaucoma.

## Introduction

Laser cyclophotocoagulation (CPC) procedures have become a common surgical method used in refractory glaucoma patients to lower intraocular pressure (IOP). The principle is to reduce aqueous humor formation by laser-assisted destruction of the ciliary body. Of the many types of CPC procedures, micropulse cyclophotocoagulation diode (MPCPC) has recently gained popularity for its efficacy and safety. In multiple previous studies, micropulse diode laser has been shown to be more selective in targeting damaged tissue and minimizing collateral thermal injury to adjacent tissues [1].

Contrary to classical cyclodiode procedures, such as continuous-wave CPC, MPCPC delivers repetitive, shorter pulses of energy with rest periods, which allows the tissue to cool between laser pulses. It effectively confines the thermal effect to the absorbing tissue, resulting in a reduced risk of postoperative complications [2, 3]. We present the first reported case of intermediary inflammation after MPCPC diode therapy.

## Case presentation

**Fig. 1:**
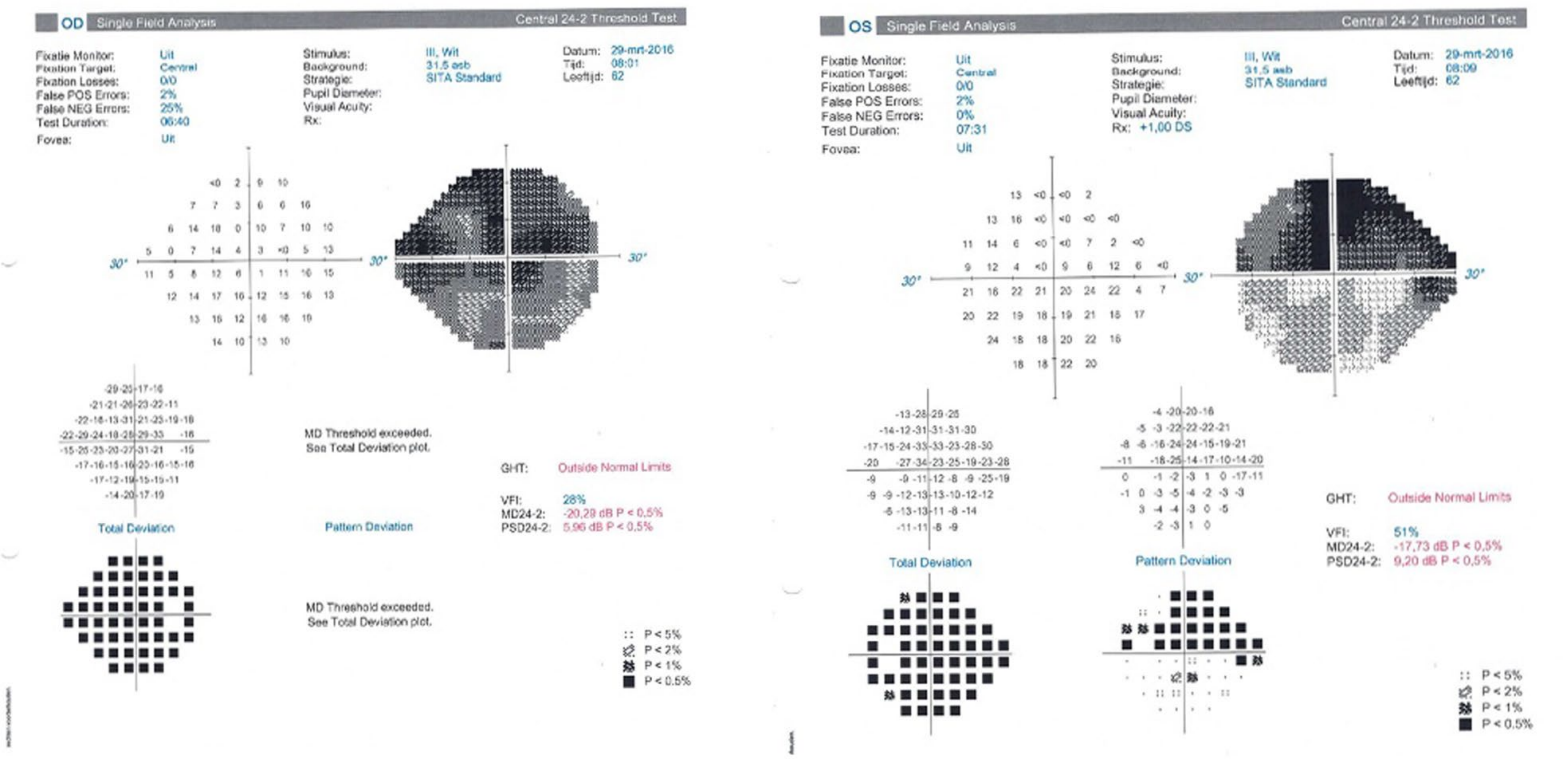
Visual fields. Right eye (RE): 24.2 SITA standard stimulus III. Left eye (LE): 24.2 SITA standard stimulus III

A 66-year-old Congolese man presented to our clinic with a very advanced primary open-angle glaucoma (POAG) that had been rapidly progressing for the past 7 years. The visual field (VF) examination showed an extensive loss, with a more pronounced visual defect in the right eye (RE) Mean Deviation (MD −20.29 dB) than in his left eye (LE) (−17.73 dB). The RE revealed an IOP of 17 mmHg and a best-corrected visual acuity (BCVA) of 0.6. In the LE, we measured an IOP of 13 mmHg and a BCVA of nearly 0.4. His topical treatment consisted of bimatoprost and brimonidine/timolol in both eyes (BE). The VFs are shown in Fig. 1.

Even though the patient did not have a serious past medical history, we discovered a positive familial POAG through his father.

A trabeculectomy with mitomycin (MMC) was performed promptly on his RE to halt the fast progression. Postoperatively, his RE was treated with suturolysis and subconjunctival 5-fluorouracil (5FU) to prevent the scarring of the bleb. One year later, a cataract operation was performed on the same eye. A mild anterior inflammation was observed 2 months after the surgery but was easily controlled with fluorometholone drops.

Unfortunately, despite topical treatment with bimatoprost and brimonidine/timolol (BE) and IOP of 11 mmHg minimum to 20 mmHg maximum (median 16 mmHg), the vision of the RE decreased to counting fingers (CF). The patient showed important hyperemia and ocular surface disease due to the topical medication. The treatment and regular follow-ups were impeded by the patient’s long and frequent travels to and from Africa.

The LE underwent a noncomplicated cataract surgery. IOP ranged from 11 to 20 mmHg (median 15.2 mmHg). Memoptic (Densmore) orally was added because of its neuroprotective properties [4]. Yttrium aluminium garnet (YAG) capsulotomy was performed in both eyes 18 months later.

The mean IOP increased slowly, as did the progression of visual field loss in his best eye (LE) despite the treatment.

**Fig. 2:**
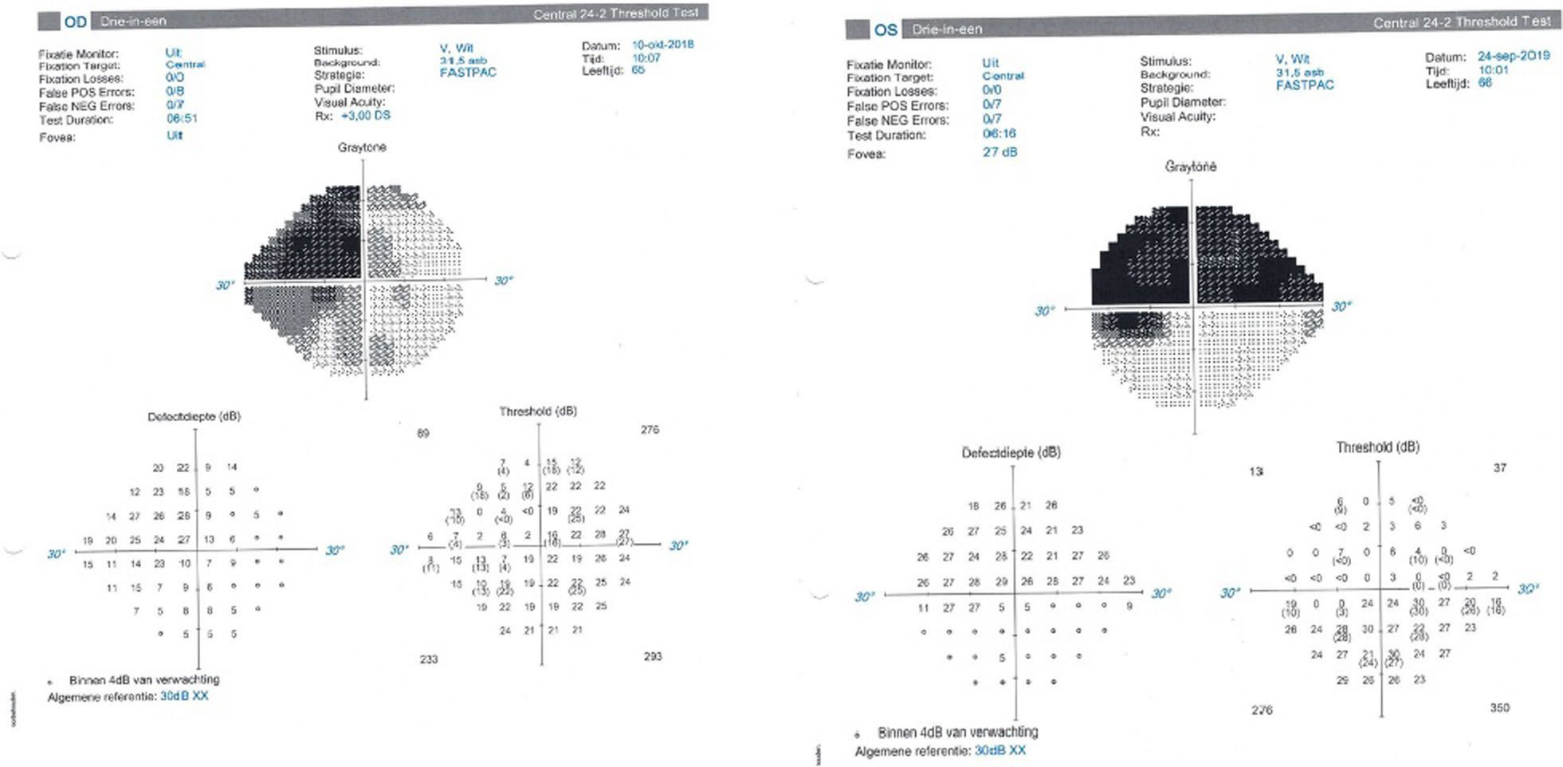
Visual fields. Right eye: 24.2 FASTPAC stimulus V. Left eye: 24.2 FASTPAC stimulus V

The last examination revealed an IOP of 20 mmHg and a BCVA of CF at 35 cm in the RE. In LE, we measured an IOP of 18 mmHg and a BCVA of 0.5. The VFs are shown in Fig. 2.

The slit lamp examination showed a calm pseudophakia and capsulotomy in BE and a nonfunctional superior trabeculectomy in the RE.

A total papillary excavation with no other fundus damage was noticeable in BE.

MPCPC was suggested as a noninvasive treatment to decrease the IOP specifically in this remaining functional eye to prevent further damage and delay a possible trabeculectomy in the LE. In the case of this patient, we were reluctant to perform a trabeculectomy because of the early failure of the trabeculectomy in his RE and the eventuality of a second failure in his last viable eye.

Because of the important ocular surface disease in our patient and the mild effect of adding a fourth hypotensive topical drop, we believed that it was not the best therapy to sufficiently decrease the mean IOP, to prevent further VF damage. According to Neelankantan *et al.* [5] and Aptel *et al.* [6], the addition of a third or fourth topical antiglaucoma medication does not reduce the IOP significantly.

Aptel *et al.* [6] demonstrated the importance of obtaining the lowest possible IOP to prevent the visual field progression. Taking into consideration the aforementioned elements, we opted for an MPCPC in this patient.

MPCPC was performed under general anesthesia (without a retrobulbar block to avoid potential hemorrhagic complications) on both eyes. The first version treatment parameters recommended by Iridex were 2000 mW of 810 nm infrared diode micropulse laser, 31.3% duty cycle (0.5 ms on-time/1.1 ms off-time), and 90 seconds of laser delivery by “swiping” each inferior and superior hemisphere. The conjunctiva was covered by a layer of hydroxypropyl methylcellulose, and a first-generation probe was used. Subconjunctival steroids, Betamethasone acetate/betamethasone sodium phosphate were injected at the end of the procedure, with no complications being reported during the operation.

Postoperative examination on day 1 revealed a mild anterior inflammation and a lower IOP (12 mmHg) under his concomitant topical treatment (bimatoprost once a day and brimonidine/timolol twice a day) in BE. In the LE, we noticed a thin inflammatory membrane attached to the posterior surface of the posterior chamber intraocular lens (PCIOL).

The vitreous was clear in the RE. In the LE, we observed a mild vitritis with a blurry image of the optic disc and retina. Topical preservative-free dexamethasone drops were added three times a day in the RE and six times a day in the LE.

During the scheduled 1-week follow-up, the patient complained of vision decrease and mild pain in his LE for the last 2 days. The examination reported a BCVA of CF 35 cm in the RE and 0.15 in the LE compared with 0.5 preoperatively. Using topical steroid drops and the usual hypotensive topical treatment, the IOP was 11 mmHg and 10 mmHg in the RE and LE, respectively.

The slit lamp examination was unchanged in the RE but it showed a diffuse punctuate epithelial keratopathy (PEK) in the LE along with a thick inflammatory membrane attached to the posterior surface of the PCIOL.

**Fig. 3:**
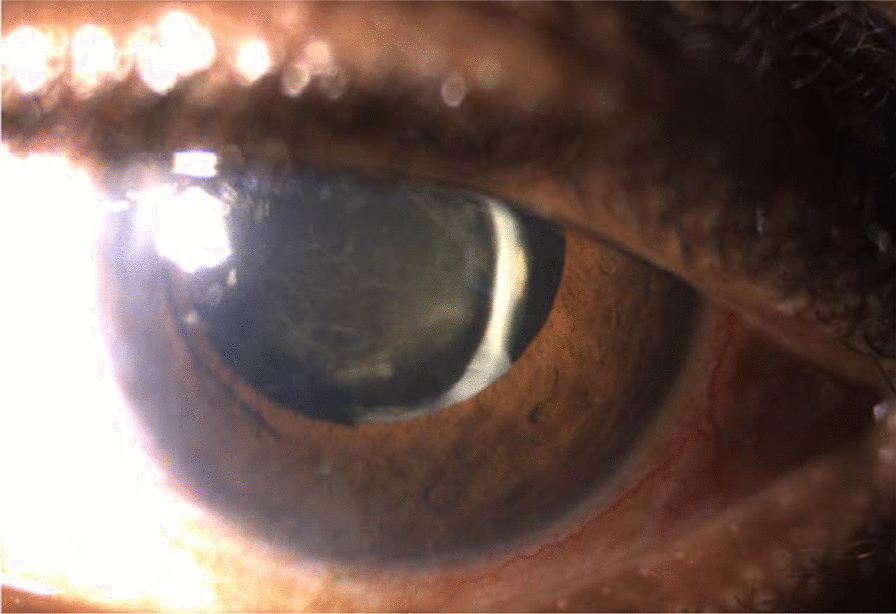
Left eye 7 days after micropulse cyclophotocoagulation diode

A moderate anterior chamber inflammation was visible, and the vitritis had progressed relatively to the prior visit. Due to this, a detailed fundoscopic examination was difficult to obtain, but we could still see a blurry image of the optic disc and the retina. There were no signs of retinal detachment nor retinitis. The subconjunctival residues of the betamethasone acetate/betamethasone sodium phosphate celestone were still substantial in both eyes. This inflammatory reaction is visible in Fig. 3.

To manage this complication, the patient was treated with a high dose of topical preservative-free dexamethasone drops once every hour combined with an oxytetracycline + hydrocortisone ointment before bedtime in the LE. The hypotensive medication was changed to carteolol to prevent any further pro-inflammatory effect by brimonidine and prostaglandins.

**Fig. 4:**
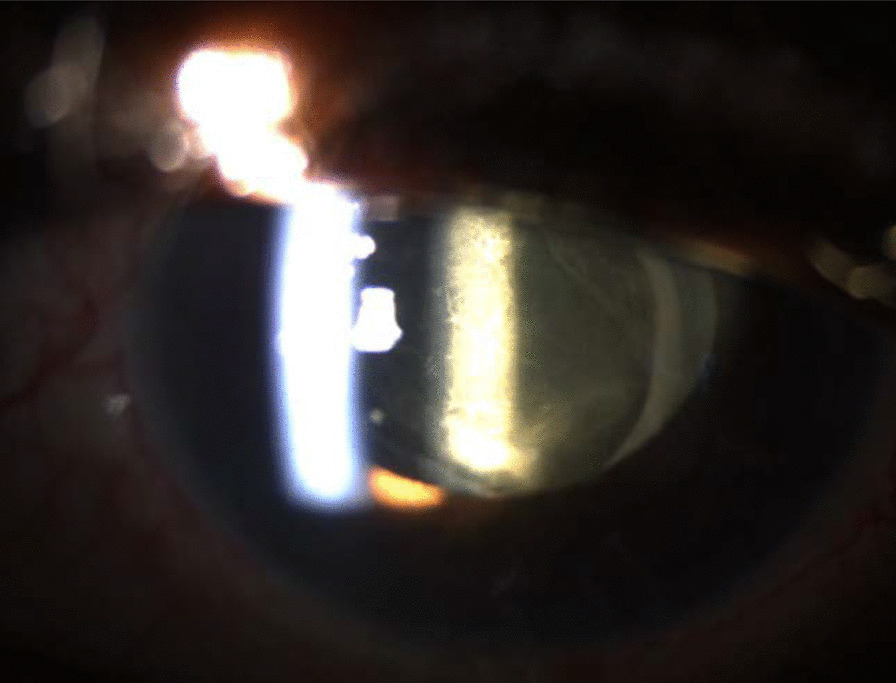
Left eye 13 days after micropulse cyclophotocoagulation diode

One week after intensive treatment, there was no clinical improvement of either the posterior inflammatory membrane, nor the vitritis, and the BCVA remained the same (Fig. 4). The IOP was of 11 mmHg in the RE (bimatoprost + brimonidine/timolol drops) and 12 mmHg in the LE (carteolol). To improve the situation and save the vision of his remaining functional eye, a vitrectomy for the LE was proposed.

The procedure allowed for a clear vitreous, free of inflammation, and the PCIOL remained stable at the end of the intervention. The usual post-vitrectomy topical treatment of combined tobramycin/dexamethasone and diclofenac, four times a day, were started in the LE. In the RE, the IOP-lowering medications (bimatoprost + brimonidine/timolol) were continued without topical corticosteroids.

Day 1 post-vitrectomy, the left BCVA was already improved to 0.2. The IOP was 12 mmHg. Slit lamp examination showed a very mild anterior reaction. The inflammatory membrane behind the intraocular lens was totally removed, and the fundus was clear, with the retina intact.

One week after the vitrectomy, examination of the RE was unchanged with a BCVA of CF 30 cm and an IOP of 7 mmHg under the same hypotensive topical three-therapy treatment.

**Fig. 5:**
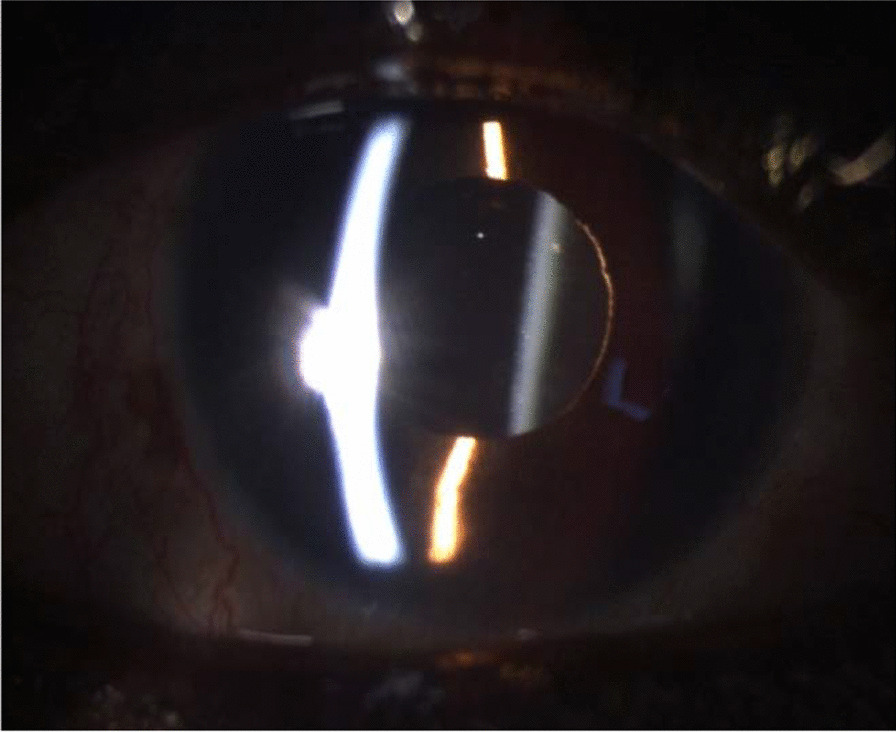
Left eye after vitrectomy

Examination of the LE revealed a BCVA of 0.1, an IOP of 28 mmHg under the same anti-inflammatory topical treatment but without hypotensive medications; slit lamp examination showed diffuse PEK, a mild residual anterior chamber reaction. As we can see in Fig. 5, the vitreous cavity was clear and the fundus was visible and stable. Bimatoprost + brimonidine/timolol drops were administered in BE to maintain low pressure. Artificial tears were given in high quantity to restore proper epithelial integrity. To avoid a new and significant inflammatory reaction, topical corticosteroids (dexamethasone) were administered in the LE, in addition to the normal postoperative treatment.

## Discussion

Our case report underlines an unexpected complication with a noninvasive method in a functionally monophthalmic, middle-aged patient There is no discussion about the choice of previous treatments. Consistent and meticulous follow-up with the patient was not possible because he resided in Kinshasa for extensive periods of time. The patient was treated to the best of our ability to stabilize the disease and within the constraints that we had to work with. Bernardi *et al.* [7], in a recent article, insist on the fact that MPCPC is a low-risk procedure, making it applicable to a broad spectrum of glaucoma cases, including patients with good central vision and not only in late-stage refractive cases.

The rapid progression of visual field loss, visual deterioration, and ocular surface disease, despite trabeculectomy in the RE, affected our decision to treat the patient before any further loss of the vision in LE. Our decision residing in our medical oath “*Primum non nocere*” made us opt for a noninvasive (or less invasive) treatment for his best eye. Despite our cautious and guarded approach, the patient had a major complication after micropulse. We were concerned about the vision in his remaining functional eye not improving after high doses of steroids. His social situation was also taken into consideration as he was a single man with little support, having to use public transportation. A vitrectomy was undergone to remove the thick inflammatory membrane from the back of his intraocular implant.

Anterior chamber inflammation, phthisis bulbi, hypotony, cystoid macular edema (CME), and scleral thinning, although at a lesser rate than continuous-wave CPC, have been previously reported. However, and to the best of our knowledge, no case of intermediary inflammation necessitating a vitrectomy as a complication of MPCPC diode has been described before. Aquino *et al.* [8] compared the efficacy and safety of MPCPC versus continuous-wave CPC in refractory glaucoma and reported a lower rate of complications in the micropulse group with a more consistent and predictable effect in lowering intraocular pressure. In their study, only 4% of cases showed prolonged inflammation after micropulse procedure compared with 30% in the continuous-wave CPC group, and the inflammation concerned only the anterior chamber. No case of intermediary inflammation has been reported. We can note the same from the conclusions of Emanuel *et al.* [9], who studied a large cohort of patients having undergone MPCPC; 86% had some degree of anterior chamber cell and/or flare at 1 week, improving to 46% at 3-month follow-up, but none of their patients (84 eyes) has had intermediary inflammatory reaction like our patient.

In the large longitudinal cohort study of Yelenskiy *et al.* [10] (197 eyes), only 2% developed postoperative cystoid macular edema as a complication. They do not report other severe complications.

Dhanireddy *et al.* [11] reported in their retrospective case series of 64 patients 2 patients with severe inflammation and hyphema post-MPCPC procedure. Again, those only concerned the anterior segment.

Even in children, no cases of intermediary uveitis have been reported. Abdelrahman *et al.* [12] studied 45 eyes of children. They proved again that the rate of complications is lower with micropulse mode. However, two eyes developed pain and anterior uveitis.

Zaarour *et al.* [13] reported in their study a lower rate of complications after MPCPC procedures compared with those of Emanuel *et al.* or Williams *et al.* In fact, they did not observe any major complications, only transient inflammatory of the anterior chamber, which did not last longer than 1 month postoperation. Zaarour *et al.* pointed out an interesting hypothesis to this fact. They included only Caucasian patients, unlike these two other studies where respectively 4% and 29% African Americans were included. The aforementioned observation could be referenced to our patient, who was African and had a significant inflammatory reaction that we had never witnessed before in other patients.

In fact, it is thought that non-white races have a higher risk of developing prolonged inflammation and hypotony resulting in decreased BVA after diode CPC and other glaucoma surgeries [1].

As a result, we have tried to focus part of our discussion on African people treated by CPC. To our knowledge, there are no other studies of MPCPC other than those reported in this paper, although we did find the study of Abdull *et al.* [14], who investigated the safety and effectiveness of continuous CPC in Nigeria (Africa). In this large cohort of 201 eyes, 11 cases of mild anterior uveitis and one case of severe uveitis have been reported as complications. They did not describe the anatomical location of “severe” uveitis, but because they clearly made the difference with the “mild anterior” uveitis, it can be assumed that they referred to an inflammatory reaction similar to our patient. Furthermore, it is important to remember that Abdull *et al.* [14] analyzed patients treated by continuous CPC and not micropulse CPC like our patient.

To our knowledge, there have been no studies investigating the correlation of the complications with different factors such as age, race, severity of the disease, number of medications, number of previous operations, energy levels delivered by the laser, or time of swiping.

## Conclusion

**Fig. 6:**
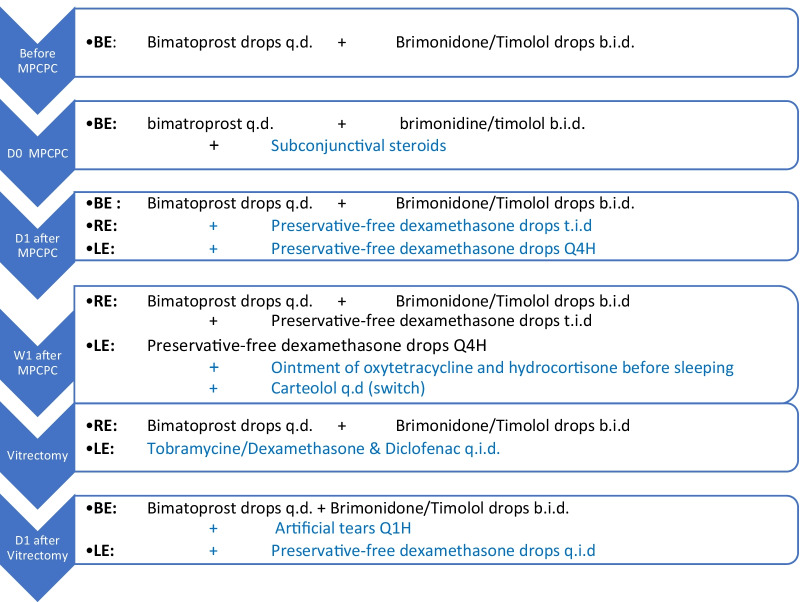
Timeline of the patient's treatment and medications

Intermediary uveitis is a rare complication after glaucoma surgery. Micropulse cyclophotocoagulation diode (MPCPC) is nowadays used increasingly and is praised for its safety and fewer complications. This case illustrates that rare, important complications such as intermediary inflammatory reaction can occur. This case is about an unexpected complication with a noninvasive surgery. It demonstrates the importance of informing patients about the possible risks and sometimes rare complications. Patient selection and regular follow-up in cases prone to important complications are crucial (Fig. 6).

## Data Availability

The datasets used and/or analyzed during the current study are available from the corresponding author on reasonable request.
